# Identification, molecular characteristics, and tissue differential expression of *DGAT2* full-CDS cDNA sequence in Binglangjiang buffalo (*Bubalus bubalis*)

**DOI:** 10.5194/aab-63-81-2020

**Published:** 2020-03-11

**Authors:** Fangting Zhou, Yongyun Zhang, Xiaohong Teng, Yongwang Miao

**Affiliations:** 1 Faculty of Animal Science and Technology, Yunnan Agricultural University, Kunming 650201, Yunnan, China; 2 Teaching Demonstration Center of the Basic Experiments of Agricultural Majors, Yunnan Agricultural University, Kunming 650201, Yunnan, China

## Abstract

It has been found that diacylglycerol acyltransferase-2 (DGAT2) plays a crucial role in the synthesis of triglycerides (TGs) in some mammals, but its role in buffalo lactation is unclear. In the present study, the *DGAT2* full-CDS cDNA sequence of Binglangjiang buffalo was isolated, and the physicochemical characteristics and structure of its encoding protein were characterized. Furthermore, the differential expressions of this gene in 10 tissues of lactating and non-lactating buffalo were analyzed by real-time quantitative PCR (RT-qPCR). The results showed that the coding region (CDS) of this gene was 1086 bp in length, encoding a peptide composed of 361 amino acid residues. The deduced amino acid sequence shared more than 98.6 % identity with that of cattle, zebu, yak, and bison in the Bovidae family. Buffalo DGAT2 protein is a slightly hydrophobic protein with a transmembrane region, which functions in membrane of endoplasmic reticulum. Besides, this protein belongs to the LPLAT_MGAT-like family and contains a conserved domain of DAGAT that has a function in the synthesis of TGs. The multi-tissue differential expression analysis demonstrated that *DGAT2* was expressed in the heart, liver, mammary gland, and muscle in both non-lactating and lactating buffalo. And its expression level in the heart, liver, and mammary gland during lactation was significantly higher than that during non-lactation. The results indicate that buffalo *DGAT2* may be involved in milk fat synthesis. This study can establish a foundation for further elucidating mechanisms of the buffalo *DGAT2* gene in milk fat synthesis.

## 1 Introduction

Diacylglycerol acyltransferase (DGAT) is an integral membrane protein located in the endoplasmic reticulum (ER), which catalyzes the covalent binding of diacylglycerol (DAG) with fatty acid acyl coenzyme A to form triglyceride (TG) (Bhatt-Wessel et al., 2018). So far, two DGAT isoforms have been identified, DGAT1 and DGAT2. Although DGAT1 and DGAT2 share similar biochemical functions and tissue expression patterns, they are significantly different. The genes encoding these two enzymes belong to different gene families, and their DNA or protein sequences are not very similar. *DGAT1* belongs to the acyl coenzyme A cholesterol acyltransferase family, while the *DGAT2* belongs to another highly conserved monoacylglycerol acyltransferase gene family (Cases et al., 2001; Lardizabal et al., 2001; McFie et al., 2011). The overexpressed *DGAT2* produced more intracellular triglycerides than the overexpressed *DGAT1* (Stone, 2004). In addition, the mice with *DGAT2* knockout died shortly after birth, but mice with *DGAT1* knockout survived (Stone, 2004). The above results suggest that DGAT2 may play a more important role in DGAT isoforms. TG is the main form of metabolic energy storage, which is stored in the hydrophobic core of cytoplasmic lipid droplets until it is needed (Walther and Farese, 2012). Previous studies have revealed that DGAT2 was a protein that was presented complexly in both ER membranes and lipid droplets, and it was co-located and interacted with various proteins to improve the efficiency of triglyceride synthesis, such as mannoside acetylglucosaminyltransferase 2 (MGAT2), stearoyl-CoA desaturase 1 (SCD1), and calnexin (Man et al., 2006; Jin et al., 2014; Brandt et al., 2019). In addition, studies have shown that loss of DGAT2 or fatty acid transport protein (FATP1) function blocked lipid droplets (LD) expansion in *Caenorhabditis elegans* (Xu et al., 2012). The FATP1–DGAT2 complex acts at the ER–LD interface and couples the synthesis and deposition of triglycerides into LDs both physically and functionally (Xu et al., 2012).

In recent years, studies on the *DGAT2* gene in some mammals have been carried out. The human *DGAT2* gene is located on chromosome 11q13.5, and it contains eight exons and seven introns. The CDS length of this gene is 1167 bp. The coding region of the mouse *DGAT2* gene is 1164 bp in length, and it also contains eight exons and seven introns (Cases et al., 2001). Bovine *DGAT2* has been mapped to BTA15q25-26 by fluorescence in situ hybridization (FISH) (Winter et al., 2003). Studies have shown that the polymorphisms of *DGAT2* gene were associated with economic traits in some domestic animals, such as carcass, meat quality, and fat yield traits in commercial feedlot steers (Li et al., 2009); back-fat thickness in pigs (Yin et al., 2012); carcass weight and shear force in domestic pigeons (Mao et al., 2018); body weight and length in cattle (Zhang et al., 2007; Mao et al., 2008); and milk yield and fat percentage in goats (An et al., 2011).

Water buffalo (*Bubalus bubalis*), as an important livestock resource in tropical and subtropical countries, has made an important contribution to the agricultural production of meat and milk as well as certain forms of labor (Mokhber et al., 2018; Du et al., 2019). Water buffalo provides more than 5 % of the world's milk supply. Its milk contains more fat, protein, lactose, and minerals than cow milk and is used to make butter, high-quality cheese, and other high-quality dairy products (Michelizzi et al., 2010). Water buffalo have leaner meat that contains less fat and cholesterol than beef while having a comparable taste (Michelizzi et al., 2010). Over the past 50 years, the total milk production of buffalo has increased significantly because its population is growing at an annual rate of 1.65 % (Du et al., 2019). There are two types of buffalo – river buffalo and swamp buffalo – which differ in chromosome number, morphology, and behavior (Nahas et al., 2013). The river buffalo is mainly used for milk production, while the swamp buffalo is mainly used for draught. Binglangjiang buffalo is an indigenous river buffalo distributed in the basin of Binglang River in western Yunnan, China. The average milk yield of this buffalo during a lactation period is 2452.2±553.8 kg with the protein and milk fat percentage of 4.60 % and 6.82 %, respectively. To date, the genes related to the milk production traits of buffalo and their mechanism of action are not clear. Previous studies have shown that the *DGAT2* gene is probably an important functional gene involved in milk fat synthesis (An et al., 2011), but there are few studies on the buffalo *DGAT2* gene. The goal of this study was to isolate and identify the *DGAT2* gene of Binglangjiang buffalo and to determine the physicochemical characteristics, structure, and interaction of the protein encoded by the gene. In addition, the differential expression of the *DGAT2* gene in 10 tissues of lactation and non-lactation animals was analyzed to evaluate whether the buffalo *DGAT2* gene plays a role in milk fat synthesis.

## 2 Materials and methods

### 2.1 Sample collection, RNA extraction, and cDNA synthesis

In accordance with the Guide for Animal Care and Use of Experimental Animals, all procedures for sample collection were performed and approved by the Yunnan Provincial Experimental Animal Management Committee. Six healthy adult female Binglangjiang buffalo (river type) were derived for sampling, including three non-lactating buffalo (dry period, about 60 d before parturition) and three lactating buffalo (mid-lactation, about 60 d postpartum). All buffalo were managed in a similar fashion. After the animals were slaughtered, the heart, liver, spleen, lung, kidney, small intestine, mammary gland, rumen, muscle, and brain were collected and preserved immediately in liquid nitrogen and then stored at -80 ${}^{\circ }$C until further processing. Total RNA for each tissue was extracted using RNAiso Plus (TaKaRa, Dalian, China) following the manufacturer's instructions. The integrity of total RNA was determined by 1.5 % agarose gel electrophoresis. Then its concentration and purity were determined using the NanoDrop 2000 UV–Vis spectrophotometer (Thermo Fisher Scientific, Waltham, MA, USA). The cDNA was synthesized from 3 µg RNA for each sample using an oligo(dT)${}_{18}$ primer and M-MLV reverse transcriptase (TaKaRa, Dalian, China).

### 2.2 Isolation of CDS sequence of the *DGAT2* gene

Based on the predicted mRNA sequence of the buffalo *DGAT2* gene (accession no. XM_006045187), a pair of primers were designed to amplify the complete coding region (CDS) of the buffalo *DGAT2* gene using Primer Premier 5.0. Detailed information about the primers is presented in Table 1.

**Table 1:** Primers used for *DGAT2* isolation and RT-qPCR.

Gene	Prime sequences (5${}^{\prime }$–3${}^{\prime }$)	Amplification	Annealing	Usage
		length (bp)	temperature (${}^{\circ }$C)	
*DGAT2*	F: TCCGCACCCCAGGCAGTA	1331	60.3	gene isolation
	R: CCCACAGACACCCATGACG			
*DGAT2*	F: GTCCTGTCTTTCCTCGTGCT	141	55.6	expression detection
	R: CCTCCTGCCACCTTTCTT			
*ACTB*	F: TGGGCATGGAATCCTG	196	57.9	internal reference
	R: GGCGCGATGATCTTGAT			

The buffalo *DGAT2* gene was isolated using mixed cDNA of various tissues as a template. PCR was performed in a volume of 20 µL containing 10 µL 2× GC Buffer I (5 mmol ${\mathrm{Mg}}^{2+}$ Plus), 3.2 µL dNTP mixture (2.5 mmol L${}^{-1}$), 0.2 µL (5 U µL${}^{-1}$) *LA Taq* polymerase (TaKaRa, Dalian, China), 0.4 µL (10 µmol L${}^{-1}$) each primer, 2 µL cDNA and 3.8 µL sterile water. PCR procedure consisted of an initial denaturation step at 94 ${}^{\circ }$C for 5 min, following by 35 cycles of denaturation at 94 ${}^{\circ }$C for 30 s, annealing at 60.3 ${}^{\circ }$C for 30 s, and extension at 72 ${}^{\circ }$C for 2 min. The final extension was conducted at 72 ${}^{\circ }$C for 5 min. The PCR products were performed by 1 % agarose gel electrophoresis and then purified with Gel Extraction Kit (OMEGA). The purified PCR products were combined with pMD18-T Vector (TaKaRa, Dalian, China) and cloned into Trans1-T1 Phage Resistant Chemically Competent Cells (TransGen Biotech Co., Ltd.) according to the manufacturer instructions. Then the target DNA fragment of recombinant plasmids were sequenced bi-directionally by Shanghai Biological Engineering Technology Services Co., Ltd. (Shanghai, China). At least 10 independent clones were sequenced.

### 2.3 Bioinformatics analysis

The raw data obtained in this study were checked, proofread, and outputted via SeqMan and EditSeq in Lasergene 7 software package (DNAStar Inc., USA). The open reading frame (ORF) was determined by ORF Finder (http://www.ncbi.nlm.nih.gov/gorf/, last access: 15 September 2019). Then, the homologous search is carried out to identify gene attribute by using the BLAST program (https://blast.ncbi.nlm.nih.gov/Blast.cgi, last access: 15 September 2019) in NCBI database. Molecular weights and theoretical isoelectric points (pI) were calculated using ProtParam tool (http://web.expasy.org/protparam/, last access: 15 September 2019). Signal peptides were predicted using the SignalP 4.1 Server (http://www.cbs.dtu.dk/services/SignalP/, last access: 15 September 2019). ProtScale (http://web.expasy.org/protscale/, last access: 15 September 2019) was used to predict the hydropathy with the relative weight of the window edges compared to the window center was 100 % and weight variation model. The transmembrane region and conserved domains were determined using TMHMM version 2.0 (http://www.cbs.dtu.dk/services/TMHMM/, last access: 15 September 2019) and NCBI server (http://www.ncbi.nlm.nih.gov/BLAST, last access: 15 September 2019). The inferred secondary structures were determined using SOPMA (http://npsa-pbil.ibcp.fr/, last access: 15 September 2019). And the parameters were four conformational states, and the similarity threshold was eight. The tertiary structure was predicted by SWISS-MODEL (http://swissmodel.expasy.org/, last access: 15 September 2019) with homologous modeling method. The subcellular localization and amino acid modifications were further predicted by ProtComp 9.0 (http://linux1.softberry.com/berry.phtml, last access: 15 September 2019) and Prosite Scan (http://prosite.expasy.org/prosite.html, last access: 15 September 2019). Protein functional analysis was conducted by InterProScan (http://www.ebi.ac.uk/interpro/search/sequence-search, last access: 15 September 2019). STRING was used to determine the interaction between proteins (https://string-db.org/, last access: 15 September 2019).

### 2.4 Differential expression analysis

The mRNA abundance of the buffalo *DGAT2* gene in 10 different tissues from lactating and non-lactating buffalo was detected by RT-qPCR. The relative expression of this gene was determined using *ACTB* gene as endogenous control. The primers designed for the RT-qPCR are listed in Table 1. The RT-qPCR was performed using iQ™5 (Bio-Rad Laboratories) with TB Green^®^ Premix Ex Taq™ II (Tli RNaseH Plus), Bulk (Takara, Dalian, China) according to the manufacturer's instructions. Each reaction mixture contained 10 µL TB Green^®^ Premix Ex Taq™ II (Tli RNaseH Plus), 0.8 µL each primer (10 Mm), 2 µL template cDNA, 6.4 µL sterile water. The program was 95 ${}^{\circ }$C for 30 s, followed by 40 cycles of 95 ${}^{\circ }$C for 5 s, 60 ${}^{\circ }$C as optimal annealing temperature for 30 s, 72 ${}^{\circ }$C for 30 s. A final melting program was carried out to create melt curves. All the samples were executed in triplicates. The relative expression levels of the gene in various tissues were evaluated using comparative method of 2${}^{-\Delta \Delta Ct}$. The average expression of each tissue for 3 lactating buffalo or 3 non-lactating buffalo was calculated to draw a bar graph. Statistical comparisons were performed by Independent – Samples T Test using SPSS 19.0 (SPSS Inc., Chicago, IL). P<0.05 was declared as significant level. P<0.01 were declared as extremely significant level.

### 2.5 Sequence similarity and phylogenetic analysis

The homologous nucleotide and amino acid sequences of buffalo DGAT2 were obtained by using the BLAST program (https://blast.ncbi.nlm.nih.gov/Blast.cgi, last access: 15 September 2019) in NCBI database. The information of homologous sequences is listed in Table S1 in the Supplement. The sequence identity of homologous proteins among species was evaluated by Megalign program with Clustal W method. ClustalX (Jeanmougin et al., 1998) was used for sequence alignment of homologous sequences by the method of doing complete alignment. Based on nucleotide sequence and amino acid sequences, a phylogenetic tree of neighbor-joining was constructed using Kimura 2-parameter model and Jones–Taylor–Thornton (JTT) model by Mega 7 software (Kumar et al., 2016), respectively. Statistical reliability of the groups within phylogenetic trees was assessed using the bootstrap method with 5000 replications.

## 3 Results

### 3.1 Isolation and identification of buffalo *DGAT2*

**Figure 1:**
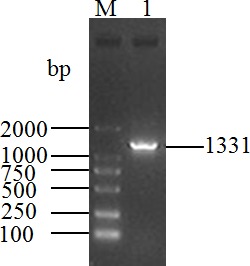
RT-PCR fragment of water buffalo *DGAT2* gene. M, Marker-DL2000; 1, sample number.

**Figure 2:**
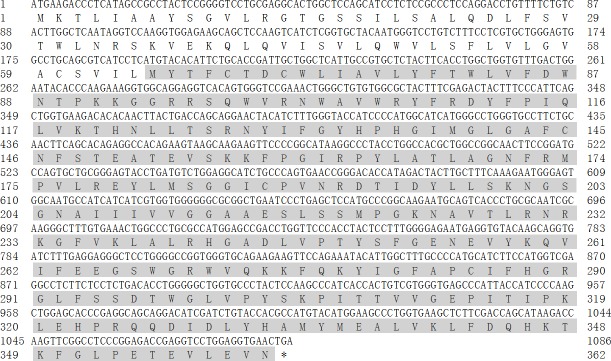
The CDS of buffalo *DGAT2* and its encoded amino acid sequence. Shaded area indicates DAGAT conserved domain (AA65-361).

The length of the PCR product obtained from the cDNA mixture of each tissue was 1331 bp (Fig. 1), which was consistent with the expected size. The obtained sequence contains a full-length 1086 bp CDS determined by ORF Finder program, which encodes a peptide composed of 361 amino acid residues. The homology search was carried out by using the BLAST program in NCBI database, and the results showed that the length of the CDS was the same as that of the *DGAT2* gene of cattle (NM_205793), zebu (XM_019975405), yak (XM_005902498), sheep (XM_027979550), and goat (NM_001314305), and the corresponding consistency was 98.9 %, 98.71 %, 98.71 %, 97.79 %, and 97.51 %, respectively. Therefore, the sequence was identified as that of buffalo *DGAT2* gene. The sequence was then submitted to the NCBI database under accession no. MK651507. The CDS of buffalo is 1086 bp and encodes a peptide composed of 361 amino acid residues. The base composition of A, G, T, and C for the CDS of buffalo *DGAT2* was 20.99 %, 27.81 %, 21.73 %, and 29.47 %, respectively, and the content of G + C was 57.27 %. The CDS and its deduced amino acids are presented in Fig. 2.

### 3.2 Sequence similarity and phylogenetic analysis

**Figure 3:**
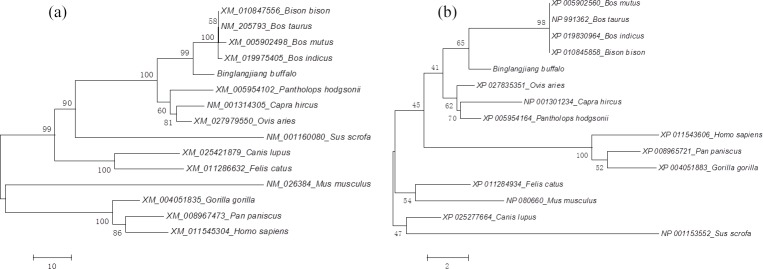
Phylogenetic tree based on DGAT2 sequences. **(a)** Phylogenetic tree based on nucleotide sequences and **(b)** phylogenetic tree based on amino acid sequences.

The identity and divergence between the buffalo DGAT2 amino sequence and its homologous sequence was analyzed. The results are shown in Fig. S1 in the Supplement. The sequence of buffalo DGAT2 was more than 98.6 % identity with that of cattle (*Bos taurus*), zebu (*Bos indicus*), yak (*Bos mutus*), and bison (*Bison bison*). Phylogenetic trees based on nucleotide and amino acid sequences all showed that the buffalo clustered with the other species in Bovidae, but in the species of Bovidae, buffalo forms a separate branch (Fig. 3a and b).

### 3.3 Basic characteristics of buffalo DGAT2

The basic physicochemical properties of buffalo DGAT2 were analyzed with cattle (accession no. NP_991362) as a control, and the results are given in Table 2. There was no significant difference in the basic physicochemical properties of DGAT2 between buffalo and cattle. The grand average of hydropathicity (GRAVY) for buffalo DGAT2 was 0.054, indicating that it was a slightly hydrophobic protein. Instability index (II) of buffalo DGAT2 is 37.59, indicating that it was a stable protein.

**Table 2:** Basic characteristics of DGAT2 for buffalo and cattle.

Characteristics	Water buffalo	Cattle
Isoelectric point (PI)	9.41	9.49
Molecular weight	40.9 KD	40.9 KD
Formula	$C_{1891}H_{2903}N_{489}O_{501}S_{14}$	$C_{1891}H_{2903}N_{489}O_{501}S_{14}$
Strongly acidic amino acids (D, E)	26	25
Strongly basic amino acids (K, R)	39	39
Polar amino acids (N, C, Q, S, T, Y)	93	95
Hydrophobic amino acids (A, I, L, F, W, V)	141	142
Instability index (II)	37.59	36.69
Grand average of hydropathicity (GRAVY)	0.054	0.066
Aliphatic index (AI)	93.91	94.71

### 3.4 Signal peptide and transmembrane helix

**Figure 4:**
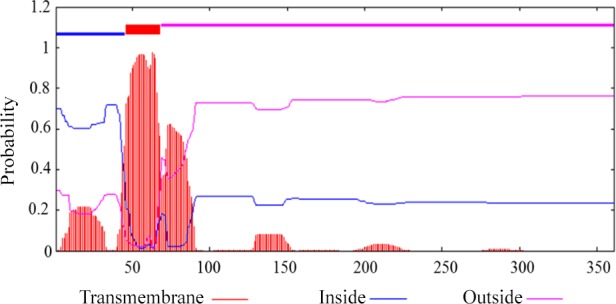
Predicted transmembrane helix in buffalo DGAT2.

Prediction showed that buffalo DGAT2 had no N-terminal signal peptide, which indicated that it was a non-secretory protein. Transmembrane analysis by TMHMM version 2.0 demonstrated that it contained a transmembrane domain (AA46-68) (Fig. 4).

### 3.5 Family and domains

**Figure 5:**
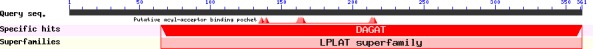
Putative conserved domain of buffalo DGAT2.

Conserved domain prediction showed that buffalo DGAT2 contained a functional domain of DAGAT (AA65-361), which is closely related to the last step of catalytic formation of triacylglycerol (TAG). DAGAT domain belongs to the common domain of the LPLAT_MGAT-like (Lysophospholipid Acyltransferases of Glycerophospholipid Biosynthesis, MGAT-like) superfamily (Fig. 5).

### 3.6 Structural analysis of buffalo DGAT2

**Figure 6:**
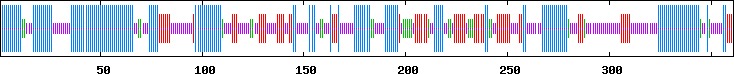
Predicted secondary structure of buffalo DGAT2. Alpha helices, extended strands, beta turns, and random coils are indicated with the longest, second longest, third longest, and shortest vertical lines, respectively.

**Figure 7:**
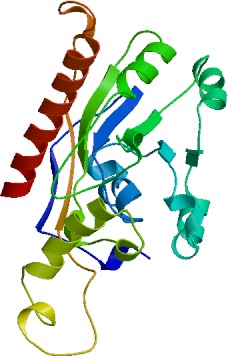
Deduced tertiary structure of buffalo DGAT2.

Secondary structure showed that DGAT2 consist of 38.50 % α-helix (139 AA), 16.9 % extended chain (61 AA), 7.76 % β turn (28 AA), and 36.84 % random coils (133 AA) (Fig. 6). The tertiary structure of buffalo DGAT2 predicted online by SWISS-MODEL based on homologous modeling is shown in Fig. 7. The sequence consistency between buffalo DGAT2 and squash (*Cucurbita moschata*) glycerol-3-phosphate (1)-acyltransferase (template: 1k30.1) was 27 %, and the coverage rate was 56 %.

### 3.7 Subcellular location and amino acid modifications

Cytoplasm/nuclear localization analysis suggested that buffalo DGAT2 was a potential endoplasmic reticulum membrane protein with a score of 10.0.

In this study, six putative kinds of modification sites were found in buffalo DGAT2 by Prosite Scan, which contained nine N-myristoylation sites, two casein kinase II phosphorylation sites, three N-glycosylation sites, four protein kinase C phosphorylation sites, one tyrosine kinase phosphorylation site, and one amidation site (Table 3).

**Table 3:** Putative functional sites in buffalo DGAT2.

Putative modification sites	Position and amino composition
N-myristoylation sites	10–15: GVlrGT; 14–19: GTgsSI; 57–62: GVacSV; 93–98: GGrrSQ; 142–147: GAfcNF; 202–207: GSgnAI; 213–218: GAaeSL; 291–296: GLfsSD; 351–356: GLpeTE
Casein kinase II phosphorylation sites	35–38: SkvE; 251–254: SfgE
N-glycosylation sites	33–36: NRSK; 146–149: NFST; 201–204: NGSG
Tyrosine kinase phosphorylation site	324–331: Rqq.DidlY
Protein kinase C phosphorylation sites	89–91: TpK; 125–127: TsR; 155–157: SkK; 228–230: TlR
Amidation site	93–96: gGRR

### 3.8 Molecular function

DGAT2 is an important member of the LPLAT superfamily, which is the acyltransferases of de novo and remodeling pathways of glycerophospholipid biosynthesis. Protein functional analysis showed that buffalo DGAT2 was an essential acyltransferase that catalyzes the formation of triglycerides from diacylglycerol.

### 3.9 Prediction and analysis of DGAT2-interacting proteins

**Figure 8:**
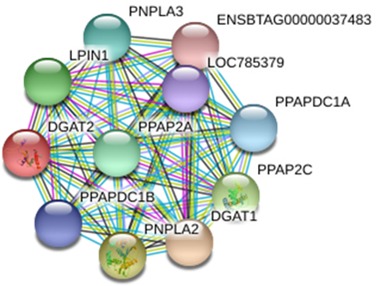
Protein–protein interaction network of DGAT2.

The protein interacting with DGAT2 was predicted by STRING database, and the protein interaction network is shown in Fig. 8. Interacting proteins include diacylglycerol O-acyltransferase 1 (DGAT1), patatin-like phospholipase domain-containing protein 2 (PNPLA2), patatin-like phospholipase domain-containing protein 3 (PNPLA3), phospholipid phosphatase 2 (PPAP2C), phosphatidic acid phosphatase type 2A (PPAP2A), lipid phosphate phosphohydrolase 1 (LPIN1), phosphatidic acid phosphatase type 2 domain-containing 1A (PPAPDC1A), phosphatidic acid phosphatase type 2 domain-containing 1B (PPAPDC1B), and an uncharacterized protein of LOC785379 and ENSBTAG00000037483.

### 3.10 Tissue expression analysis

**Figure 9:**
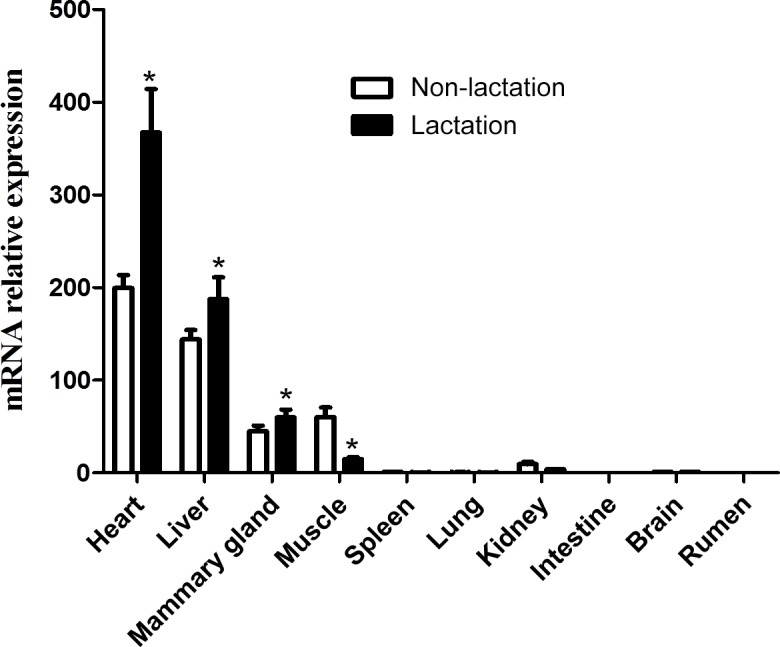
Tissue expression profile of buffalo *DGAT2* gene.

The expression of the *DGAT2* gene in 10 tissues of lactating and non-lactating buffalo was detected by RT-qPCR. Data information for tissue differential expression of buffalo *DGAT2* gene is listed in Table S2. In non-lactating and lactating buffalo, the *DGAT2* gene was found to be expressed in the heart, liver, mammary gland, muscle, and kidney, whereas almost no expression was in the spleen, lung, brain, rumen, and intestine (Fig. 9). The *DGAT2* expression in the heart, liver, and mammary gland in the lactation stage was significantly higher than that in the non-lactation stage (P<0.05), but its expression in muscle was lower in the lactation stage than in the non-lactation stage (P<0.05; Fig. 9).

## 4 Discussion

In this study, *DGAT2* gene was isolated and characterized from Binglangjiang water buffalo. The full-length CDS of this gene is 1086 nucleotides, encoding 361 amino acids, which was consistent with that of cattle (Winter et al., 2003). The basic physicochemical properties of buffalo DGAT2 were similar to those of cattle, and the amino acid sequence of buffalo DGAT2 showed high identity with other species. Phylogenetic analysis based on nucleotide and amino acid sequences both showed that water buffalo and other species of Bovidae clustered together. These results indicate that this protein is highly conservative, and buffalo DGAT2 may have a similar genetic function with other mammals, particularly the species in the family of Bovidae.

Murine DGAT2 has two transmembrane domains located near the N-terminus of the protein, and both the N- and C-termini of DGAT2 are exposed to the cytosol (Stone et al., 2006). DGAT2 deletion mutation assay in 293T cells indicates that the interaction with MGAT2 depends on the two transmembrane regions of DGAT2 (Jin et al., 2014). However, the targeting signal of DGAT2 exists in the first transmembrane region. The first transmembrane domain, but not the second transmembrane region, was sufficient for DGAT2 localization to the ER (McFie et al., 2011). In our study, one transmembrane domain was predicted in buffalo DGAT2, and this protein was predicted to locate in ER. ER is the main site of intracellular triglyceride synthesis (Bhatt-Wessel et al., 2018). We speculate that this transmembrane region may play a more important role in the process of binding to ER to realize the function of DGAT2 protein. Conservative domain prediction showed that water buffalo DGAT2 contained a functional domain of DAGAT, which belongs to the members of the LPLAT superfamily (Marchler-Bauer et al., 2017). And functional prediction showed that buffalo DGAT2 was an essential acyltransferase that catalyzes the formation of triglycerides from diacylglycerol. Previous studies have shown that diacylglycerol acyltransferase can catalyze the final step of TG formation (Sorger and Daum, 2002). Based on the above prediction results, we speculate that DGAT2 also plays this role in the ER in water buffalo.

In the present study, a number of DGAT2-interacting proteins were predicted which were involved in cell energy metabolism and lipid synthesis. Studies have revealed that DGAT1 is related to milk production traits in some species (Martin et al., 2018; Pacheco-Pappenheim et al., 2019). PNPLA2 and PNPLA3 catalyze the first step in the hydrolysis of triglycerides in adipose tissue (Murugesan et al., 2013). Studies in humans have shown that the mutation of *PNPLA2* gene leads to neutral lipid storage disease with myopathy or triglyceride deposition cardiomyopathy (Kaneko et al., 2014). LPIN1 catalyzes the formation of diacylglycerol from phosphatidic acid (PA) (Bi et al., 2015). Diacylglycerol is a direct precursor for the synthesis of neutral phospholipids, phosphatidylcholine, and phosphatidylethanolamine. PPAP2C and PPAP2A belong to the phosphatase family related to PA-phosphatase, which are responsible for the conversion of phosphatidic acid (PA) to diacylglycerol. In addition, they hydrolyze lysophosphatidic acid (LPA), ceramide-1-phosphate (C-1-P), and sphingomyelin-1-phosphate (S-1-P) (Busnelli et al., 2018). In view of the fact that the above proteins are involved in the process of lipid metabolism, we speculate that buffalo DGAT2 may be involved in the synthesis of triglycerides in the mammary gland through the interaction with these proteins. However, the interaction between DGAT2 and these proteins in buffalo mammary gland needs to be further studied.

Previous studies in some mammals have shown that *DGAT2* gene plays an important role in the process of TG synthesis, and it is also considered to be a candidate gene affecting milk yield and fat content in goats (Man et al., 2006; An et al., 2011; Jin et al., 2014). In mice and humans, *DGAT2* was found to express with the highest expression levels in the tissues of adipose tissue, liver, small intestine, and mammary gland, which are associated with TG synthesis (Cases et al., 2001). The study in yak showed that the *DGAT2* was abundant mostly in subcutaneous fat, moderate in liver, heart, longissimus dorsi, and abomasum but lower in the large intestine, small intestine, mammary gland, lungs, kidney, spleen, and rumen (Hu et al., 2019). The *DGAT2* gene was also found extensively expressed in adult pigs with the highest level in the liver (Yin et al., 2012). In this study, *DGAT2* gene was found to express in some active lipid metabolic tissues such as the heart, liver, mammary gland, and muscle of both non-lactating and lactating buffalo, indicating that the *DGAT2* gene was involved in TG synthesis in these tissues. The expression pattern of *DGAT2* in buffalo here is different from that of other species that have been studied in recent years. It is worth noting that the expression level of *DGAT2* gene in the mammary gland in the lactating stage is significantly higher than that in the non-lactating stage. During lactation stage, more TGs need to be synthesized in mammary gland to supply the need for milk fat production, so the expression of *DGAT2* is also higher than that in the non-lactation stage. This study also found that the expression of *DGAT2 *gene in the liver and heart of lactating buffalo was significantly higher than that of non-lactating buffalo, which may be related to the different physiological states of these two stages, especially the contribution of liver to TG supply of mammary gland in the lactating stage. Studies in adult mouse found that inactivation of DGAT2 in heart can result in moderate inhibition of TG synthesis and transformation (Roe et al., 2018), which revealed that *DGAT2* gene played an important role in heart in mouse. The results of this study showed that the buffalo *DGAT2* gene was highly expressed in the heart in both lactating and non-lactating buffalo, especially during the lactating period, suggesting that this gene may be related to the TG synthesis in buffalo heart, and the TG synthesis of lactating buffalo heart may be more active.

Most protein functions are regulated by the modification of some amino acids in the polypeptide chain (Song et al., 2013). Six putative kinds of modification sites were found in buffalo DGAT2 by Prosite Scan, which contained nine N-myristoylation sites, two casein kinase II phosphorylation sites, three N-glycosylation sites, four protein kinase C phosphorylation sites, one tyrosine kinase phosphorylation site, and one amidation site. Whether these putative functional sites play crucial roles in post-transcriptional level regulation for DGAT2 protein remains to be investigated further.

## 5 Conclusions

In this study, the *DGAT2* gene was isolated and characterized in Binglangjiang buffalo. The results indicate that buffalo DGAT2 is involved in catalyzing the covalent binding of diacylglycerol to fatty acid acyl coenzyme A to form TG. This function is the similar to bovine species. This gene manifested differential expression in 10 tissues during lactation and non-lactation. Compared to the non-lactating stage, the relative mRNA abundance of this gene in the lactating stage remarkably increased in the heart, liver, and mammary gland, which indicates that the gene plays an important role in these tissues. As far as the mammary gland is concerned, the *DGAT2* gene of buffalo may play an important role in milk fat synthesis during lactation, but this is produced under the limited number of samples in this study, which needs to be confirmed by further increasing the number of samples. In addition, the mechanism of buffalo *DGAT2* gene on milk production traits is still unclear, which needs to be further studied. This study will provide a foundation for further insights into the mechanism of buffalo *DGAT2* gene on milk fat synthesis.

## Supplement

10.5194/aab-63-81-2020-supplement The supplement related to this article is available online at: https://doi.org/10.5194/aab-63-81-2020-supplement.

## Data Availability

The original data of the paper are available from the corresponding author upon request.

## References

[bib1.bib1] An XP, Song SG, Hou JX, Zhu CM, Peng JX, Liu XQ, Liu HY, Xiao WP, Zhao HP, Bai L, Wang JG, Song YX, Cao BY (2011). Polymorphism identification in goat DGAT2 gene and association analysis with milk yield and fat percentage. Small Ruminant Res.

[bib1.bib2] Bhatt-Wessel B, Jordan TW, Miller JH, Peng L (2018). Role of DGAT enzymes in triacylglycerol metabolism. Arch Biochem Biophys.

[bib1.bib3] Bi L, Jiang Z, Zhou J (2015). The role of lipin-1 in the pathogenesis of alcoholic fatty liver. Alcohol Alcoholism.

[bib1.bib4] Brandt C, McFie PJ, Vu H, Chumala P, Katselis GS, Stone SJ (2019). Identification of calnexin as a diacylglycerol acyltransferase-2 interacting protein. Plos One.

[bib1.bib5] Busnelli M, Manzini S, Parolini C, Escalante-Alcalde D, Chiesa G (2018). Lipid phosphate phosphatase 3 in vascular pathophysiology. Atherosclerosis.

[bib1.bib6] Cases S, Stone SJ, Zhou P, Yen E, Tow B, Lardizabal KD, Voelker T, Farese RV (2001). Cloning of DGAT2, a second mammalian diacylglycerol acyltransferase, and related family members. J Biol Chem.

[bib1.bib7] Du C, Deng TX, Zhou Y, Ghanem N, Hua GH (2019). Bioinformatics analysis of candidate genes for milk production traits in water buffalo (Bubalus bubalis). Trop Anim Health Pro.

[bib1.bib8] Hu J, Shi B, Xie J, Zhou H, Wang J, Liu X, Li S, Zhao Z, Luo Y (2019). Tissue expression and variation of the DGAT2 gene and its effect on carcass and meat quality traits in yak. Animals.

[bib1.bib9] Jeanmougin F, Thompson JD, Gouy M, Higgins DG, Gibson TJ (1998). Multiple sequence alignment with Clustal X. Trends Biochem Sci.

[bib1.bib10] Jin Y, McFie PJ, Banman SL, Brandt C, Stone SJ (2014). Diacylglycerol acyltransferase-2 (DGAT2) and monoacylglycerol acyltransferase-2 (MGAT2) interact to promote triacylglycerol synthesis. J Biol Chem.

[bib1.bib11] Kaneko K, Kuroda H, Izumi R, Tateyama M, Kato M, Sugimura K, Sakata Y, Ikeda Y, Hirano K, Aoki M (2014). A novel mutation in PNPLA2 causes neutral lipid storage disease with myopathy and triglyceride deposit cardiomyovasculopathy: A case report and literature review. Neuromuscular Disord.

[bib1.bib12] Kumar S, Stecher G, Tamura K (2016). MEGA7: Molecular Evolutionary Genetics Analysis Version 7.0 for Bigger Datasets. Mol Biol Evol.

[bib1.bib13] Lardizabal KD, Mai JT, Wagner NW, Wyrick A, Voelker T, Hawkins DJ (2001). DGAT2 is a new diacylglycerol acyltransferase gene family: Purification, cloning, and expression in insect cells of two polypeptides from Mortierella ramanniana with diacylglycerol acyltransferase activity. J Biol Chem.

[bib1.bib14] Li J, Xu X, Zhang Q, Wang X, Deng G, Fang X, Gao X, Ren H, Xua S (2009). Association between single nucleotide polymorphisms in the dgat2 gene and beef carcass and quality traits in commercial feedlot steers. Asian Austral J Anim.

[bib1.bib15] Man WC, Miyazaki M, Chu K, Ntambi J (2006). Colocalization of SCD1 and DGAT2: Implying preference for endogenous monounsaturated fatty acids in triglyceride synthesis. J Lipid Res.

[bib1.bib16] Mao HG, Dong XY, Cao HY, Xu NY, Yin ZZ (2018). Association of DGAT2 gene polymorphisms with carcass and meat quality traits in domestic pigeons (Columba livia). Br Poult Sci.

[bib1.bib17] Mao HX, Chen H, Chen FY, Zhang CL, Wang XZ, Wang JQ (2008). Association of polymorphisms of DGAT2 gene with growth traits in Jiaxian red cattle. Yi Chuan.

[bib1.bib18] Marchler-Bauer A, Bo Y, Han L, He J, Lanczycki CJ, Lu S, Chitsaz F, Derbyshire MK, Geer RC, Gonzales NR, Gwadz M, Hurwitz DI, Lu F, Marchler GH, Song JS, Thanki N, Wang Z, Yamashita RA, Zhang D, Zheng C, Geer LY, Bryant SH (2017). CDD/SPARCLE: functional classification of proteins via subfamily domain architectures. Nucleic Acids Res.

[bib1.bib19] Martin P, Palhiere I, Maroteau C, Bardou P, Canale-Tabet K, Sarry J, Woloszyn F, Bertrand-Michel J, Racke I, Besir H, Rupp R, Tosser-Klopp G (2018). Author Correction: A genome scan for milk production traits in dairy goats reveals two new mutations in Dgat1 reducing milk fat content. Sci Rep-UK.

[bib1.bib20] McFie PJ, Banman SL, Kary S, Stone SJ (2011). Murine diacylglycerol acyltransferase-2 (DGAT2) can catalyze triacylglycerol synthesis and promote lipid droplet formation independent of its localization to the endoplasmic reticulum. J Biol Chem.

[bib1.bib21] Michelizzi VN, Dodson MV, Pan Z, Amaral MEJ, Michal JJ, McLean DJ, Womack JE, Jiang Z (2010). Water buffalo genome science comes of age. Int J Biol Sci.

[bib1.bib22] Mokhber M, Moradi-Shahrbabak M, Sadeghi M, Moradi-Shahrbabak H, Stella A, Nicolzzi E, Rahmaninia J, Williams JL (2018). A genome-wide scan for signatures of selection in Azeri and Khuzestani buffalo breeds. BMC Genomics.

[bib1.bib23] Murugesan S, Goldberg EB, Dou E, Brown WJ (2013). Identification of diverse lipid droplet targeting motifs in the PNPLA family of triglyceride lipases. Plos One.

[bib1.bib24] Nahas SM, Bibars MA, Taha DA (2013). Genetic characterization of Egyptian buffalo CSN3 gene. J Genet Eng Biotechnol.

[bib1.bib25] Pacheco-Pappenheim S, Yener S, van Valenberg H, Tzompa-Sosa DA, Bovenhuis H (2019). The DGAT1 K232A polymorphism and feeding modify milk fat triacylglycerol composition. J Dairy Sci.

[bib1.bib26] Roe ND, Handzlik MK, Li T, Tian R (2018). The Role of Diacylglycerol Acyltransferase (DGAT) 1 and 2 in Cardiac Metabolism and Function. Sci Rep-UK.

[bib1.bib27] Song S, Huo JL, Li DL, Yuan YY, Yuan F, Miao YW (2013). Molecular cloning, sequence characterization, and gene expression profiling of a novel water buffalo (Bubalus bubalis) gene, AGPAT6. Genet Mol Res.

[bib1.bib28] Sorger D, Daum G (2002). Synthesis of triacylglycerols by the acyl-coenzyme A:diacyl-glycerol acyltransferase Dga1p in lipid particles of the yeast Saccharomyces cerevisiae. J Bacteriol.

[bib1.bib29] Stone SJ (2004). Lipopenia and skin barrier abnormalities in DGAT2-deficient mice. J Biol Chem.

[bib1.bib30] Stone SJ, Levin MC, Farese RV (2006). Membrane topology and identification of key functional amino acid residues of murine Acyl-CoA:diacylglycerol acyltransferase-2. J Biol Chem.

[bib1.bib31] Walther TC, Farese RV (2012). Lipid droplets and cellular lipid metabolism. Annu Rev Biochem.

[bib1.bib32] Winter A, van Eckeveld M, Bininda-Emonds OR, Habermann FA, Fries R (2003). Genomic organization of the DGAT2/MOGAT gene family in cattle (Bos taurus) and other mammals. Cytogenet Genome Res.

[bib1.bib33] Xu N, Zhang SO, Cole RA, McKinney SA, Guo F, Haas JT, Bobba S, Farese RJ, Mak HY (2012). The FATP1-DGAT2 complex facilitates lipid droplet expansion at the ER-lipid droplet interface. J Cell Biol.

[bib1.bib34] Yin Q, Yang H, Han X, Fan B, Liu B (2012). Isolation, mapping, SNP detection and association with backfat traits of the porcine CTNNBL1 and DGAT2 genes. Mol Biol Rep.

[bib1.bib35] Zhang ZF, Chen H, Li QL, Lei CZ, Zhang CL, Wang XZ, Wang JQ, Wang YM (2007). Polymorphisms of DGAT2 gene and its associations with several growth traits in Nanyang cattle. Yi Chuan.

