# Supplementary material for: Identification, molecular characteristics, and tissue differential expression of DGAT2 full-CDS cDNA sequence in Binglangjiang buffalo (Bubalus bubalis)
Source: Arch Anim Breed. 2020 Mar 11;63(1):81–90. doi: 10.5194/aab-63-81-2020 (PMC7096739; doi:10.5194/aab-63-81-2020)
Supplement: The supplement related to this article is available online at: https://doi.org/10.5194/aab-63-81-2020-supplement. [file aab-63-81-supplement.pdf]

Supplement of Arch. Anim. Breed., 63, 81–90, 2020
https://doi.org/10.5194/aab-63-81-2020-supplement
© Author(s) 2020. This work is distributed under
the Creative Commons Attribution 4.0 License.

Open Access
Archives Animal Breeding

Supplement of

Identification, molecular characteristics, and tissue differential expression of *DGAT2* full-CDS cDNA sequence in Binglangjiang buffalo (*Bubalus bubalis*)

Fangting Zhou et al.

Correspondence to: Yongwang Miao (yongwangmiao1@126.com)

The copyright of individual parts of the supplement might differ from the CC BY 4.0 License.

Table S1. Information of homologous sequences of DGAT2 for sequence similarity and phylogenetic analysis

sequence type	species	GenBank accession number	data sources	length/bp
coding sequences	*Mus musculus*	NM_026384	NCBI database	1167
	Bos taurus	NM_205793		1086
	Sus scrofa	NM_001160080		1086
	Capra hircus	NM_001314305		1086
	Gorilla gorilla	XM_004051835		1167
	Bos mutus	XM_005902498		1086
	Pan paniscus	XM_008967473		1116
	Bison bison	XM_010847556		1086
	Felis catus	XM_011286632		1086
	Homo sapiens	XM_011545304		1077
	Bos indicus	XM_019975405		1086
	Canis lupus	XM_025421879		1086
	Ovis aries	XM_027979550		1086
	Pantholops hodgsonii	XM_005954102		1086
amino acid sequences	*Mus musculus*	NP_080660		388
	Bos taurus	NP_991362		361
	Sus scrofa	NP_001153552		361
	Capra hircus	NP_001301234		361
	Gorilla gorilla	XP_004051883		388
	Bos mutus	XP_005902560		361
	Pan paniscus	XP_008965721		371
	Bison bison	XP_010845858		361
	Felis catus	XP_011284934		361
	Homo sapiens	XP_011543606		358
	Bos indicus	XP_019830964		361
	Canis lupus	XP_025277664		361
	Ovis aries	XP_027835351		361
	Pantholops hodgsonii	XP_005954164		361

Table S2. Data information for tissue differential expression of buffalo *DGAT2* gene

tissues	relative expression levels (mean ± standard error)		*P* values
	non-lactation	lactation	
heart	199.38 ± 14.04	367.40 ± 46.71	0.04
liver	143.93 ± 10.30	187.53 ± 23.35	0.04
mammary gland	45.00 ± 6.00	60.00 ± 8.23	0.04
muscle	60.11 ± 10.45	14.97 ± 1.87	0.02
spleen	0.97 ± 0.02	0.37 ± 0.08	0.05
lung	0.82 ± 0.31	0.36 ± 0.02	0.11
kidney	9.41 ± 1.65	4.00 ± 0.12	0.01
intestine	0.09 ± 0.02	0.17 ± 0.03	0.01
brain	1.01 ± 0.19	1.01 ± 0.14	0.99
rumen	0.18 ± 0.01	0.25 ± 0.02	0.02

Note: The values are the average values for each tissue in 3 lactating buffaloes or 3 non-lactating buffaloes.

identity percent

divergence

	1	2	3	4	5	6	7	8	9	10	11	12	13	14	15		
**1**		98.6	98.6	98.6	98.6	98.3	98.9	94.5	97.8	95.6	98.1	97.8	98.9	97.8	97.8	**1**	*Bubalus bubalis*
**2**	1.4		100.0	100.0	100.0	97.5	98.1	93.6	97.0	95.0	97.8	97.0	98.1	97.0	97.0	**2**	*Bos taurus*
**3**	1.4	0.0		100.0	100.0	97.5	98.1	93.6	97.0	95.0	97.8	97.0	98.1	97.0	97.0	**3**	*Bos indicus*
**4**	1.5	0.0	0.0		100.0	97.4	98.0	96.8	96.8	94.8	97.7	96.8	98.0	96.8	96.8	**4**	*Bos mutus*
**5**	1.4	0.0	0.0	0.0		97.5	98.1	93.6	97.0	95.0	97.8	97.0	98.1	97.0	97.0	**5**	*Bison bison bison*
**6**	1.7	2.5	2.5	2.6	2.5		98.9	94.5	97.2	94.5	97.5	97.8	98.9	97.8	96.7	**6**	*Capra hircus*
**7**	1.1	2.0	2.0	2.0	2.0	1.1		95.3	97.8	95.3	98.1	98.6	99.4	98.6	97.2	**7**	*Ovis aries*
**8**	5.8	6.7	6.7	3.2	6.7	5.8	4.9		94.7	91.4	94.7	94.9	95.0	94.9	92.2	**8**	*Pan paniscus*
**9**	2.3	3.1	3.1	3.2	3.1	2.8	2.3	5.5		95.0	98.6	98.1	97.8	98.1	98.1	**9**	*Felis catus*
**10**	4.6	5.2	5.2	5.4	5.2	5.8	4.9	9.1	5.2		96.1	94.7	95.0	94.7	95.0	**10**	*Sus scrofa*
**11**	2.0	2.3	2.3	2.3	2.3	2.5	2.0	5.5	1.4	4.0		98.1	98.1	98.1	97.8	**11**	*Canis lupus dingo*
**12**	2.3	3.1	3.1	3.2	3.1	2.3	1.4	5.3	2.0	5.5	2.0		98.3	100.0	95.1	**12**	*Gorilla gorilla gorilla*
**13**	1.1	2.0	2.0	2.0	2.0	1.1	0.6	5.2	2.3	5.2	2.0	1.7		98.3	97.2	**13**	*Pantholops hodgsonii*
**14**	2.3	3.1	3.1	3.2	3.1	2.3	1.4	5.3	2.0	5.5	2.0	0.0	1.7		95.1	**14**	*Homo sapiens*
**15**	2.3	3.1	3.1	3.2	3.1	3.4	2.8	8.3	2.0	5.2	2.3	5.1	2.8	5.1		**15**	*Mus musculus*
	**1**	**2**	**3**	**4**	**5**	**6**	**7**	**8**	**9**	**10**	**11**	**12**	**13**	**14**	**15**		

Figure S1. Identity and divergence percentage of DGAT2 amino acid sequences among some species. The values above diagonal line represent the identity percentage, and the values below the diagonal represent divergence
